# Supplementary figures and images for: A comparative analysis of 24-hour movement behaviors features using different accelerometer metrics in adults: Implications for guideline compliance and associations with cardiometabolic health
Source: PLoS One. 2024 Sep 17;19(9):e0309931. doi: 10.1371/journal.pone.0309931 (PMC11407674; doi:10.1371/journal.pone.0309931)

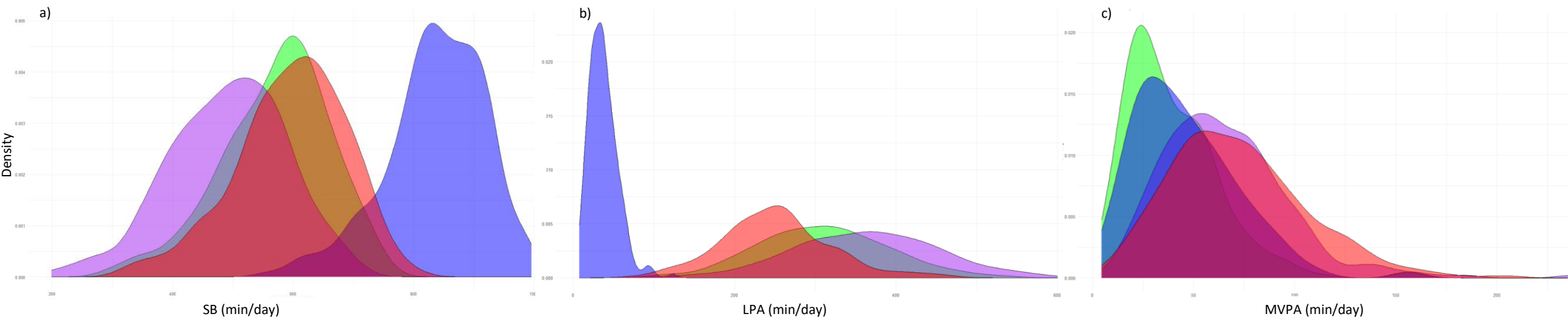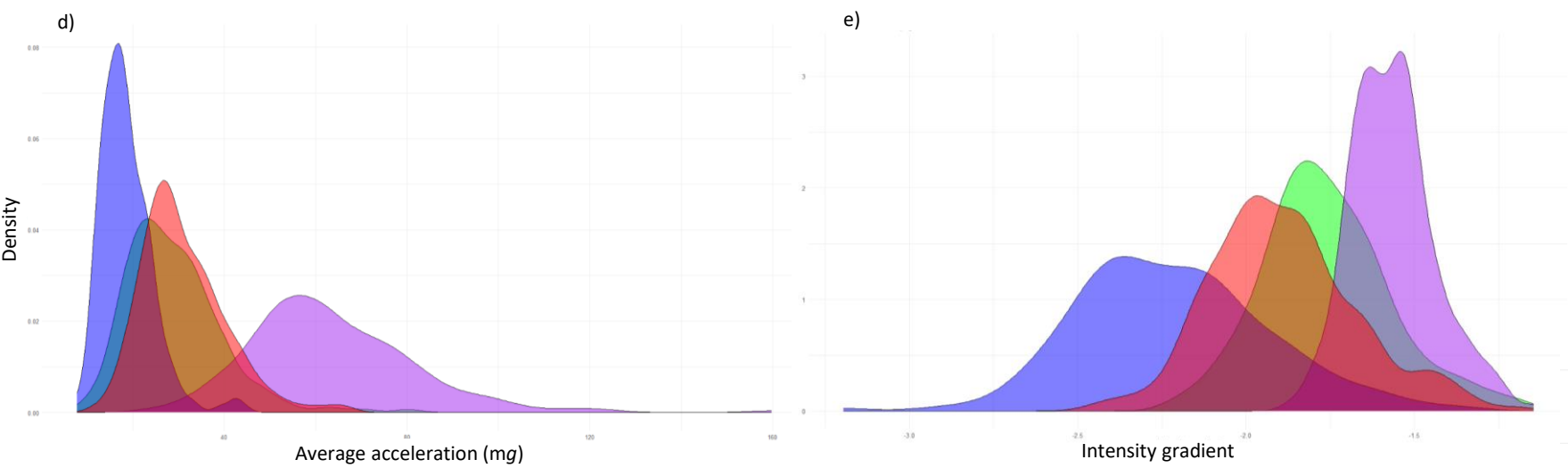

Supplement: S1 Fig — SB: sedentary behavior, LPA: light physical activity, MVPA: moderate to vigorous physical activity, ENMO: Euclidian Norm Minus One, MAD: Mean Amplitude Deviation, CPM VA: Counts Per minute Vertical Axis, CPM VM: Counts Per Minute Vector Magnitude. (PDF) [file pone.0309931.s001.pdf]
