# Supplementary material for: A comparative analysis of 24-hour movement behaviors features using different accelerometer metrics in adults: Implications for guideline compliance and associations with cardiometabolic health
Source: PLoS One. 2024 Sep 17;19(9):e0309931. doi: 10.1371/journal.pone.0309931 (PMC11407674; doi:10.1371/journal.pone.0309931)
Supplement: S1 Table — (DOCX) [file pone.0309931.s002.docx]

S1. Table with accelerometer data processing metrics used to analyze movement behavior features

| **Resultant vector/Euclidian Norm** | Square root of the sum of squared activity counts from the three axes (x, y, z) out of a tri-axial accelerometer.  $r_{i}=\sqrt{x_{i}^{2}+y_{i}^{2}+z_{i}^{2}}$ |
| --- | --- |
| **Euclidian Norm Minus One (ENMO)** | This is computed by removing one gravitational unit (static component gravity) from the resultant vector (dynamic component) using an magnitude approach. Magnitude approach: metric makes an assumption about the magnitude of the gravitational acceleration component (one gravitational unit).  $ENMO= r_{i}-1g (or 1000 mg)$  With negative ENMO values are rounded to zero.  *Specification GGIR: do.enmo=TRUE, acc.metric=“ENMO”* |
| **Mean Amplitude Deviation (MAD)** | Mean value of the dynamic acceleration component. This is computed from the resultant vector by removing the static component (gravity) from the dynamic component (deviations in velocity) using a frequency approach. Frequency approach: the metric makes an assumption about the frequency content of the gravitational acceleration component.  $MAD= \frac{1}{n}\left\vert r_{i}-\bar{r} \right\vert$  Absolute difference between Euclidian Norm and epoch level average of the Euclidian norm ($\bar{r}$). With n representing the number of samples in epoch.  *Specification GGIR: do.mad=TRUE, acc.metric=”MAD”* |
| **Counts Per Minute (CPM)** | Algorithm to calculate counts as data processing technique of Software Actilife is decribed in Neishabouri et al. 2022.  **Vertical Axis (VA)**  Summing the 1s epoch counts data for 60 sec intervals of the vertical axis (y-axis)  *Specification GGIR: do.neishabouricounts=TRUE, acc.metric= “neishabouriCount_y”*  **Vector Magnitude (VM)**  Square root of the sum of squared activity counts from three axes, i.e. resultant vetor r_i_  *Specification GGIR: do.neishabouricounts=TRUE, acc.metric= “neishabouriCount_vm”* |
