# Supplementary material for: A comparative analysis of 24-hour movement behaviors features using different accelerometer metrics in adults: Implications for guideline compliance and associations with cardiometabolic health
Source: PLoS One. 2024 Sep 17;19(9):e0309931. doi: 10.1371/journal.pone.0309931 (PMC11407674; doi:10.1371/journal.pone.0309931)
Supplement: S1 File — This document contains a more in-depth analysis to explore the impact of the metric on the associations between the cut-point dependent movement behaviors features (time use estimates in SB, LPA, MVPA) and the cut-point independent movement behaviors (average acceleration, intensity gradient) on one hand and the cardiometabolic variables on the other hand. (DOCX) [file pone.0309931.s007.docx]

## Associations between movement behaviors features and cardiometabolic variables

The choice of data processing metric has an impact on the time spent in movement behavior features, average acceleration and intensity gradient. This supplementary information shows the impact of data processing metric use on the associations between 24h-MBs compositions, average acceleration, intensity gradient (processed by four different metrics) and cardiometabolic variables. Because the data are cross-sectional causality cannot be inferred.

**Cut-point dependent movement behaviors features**

The time spent on 24h-MBs was significantly associated with BMI and HbA1c when ENMO was used (BMI: F= 3.23, p=0.02; HbA1c: F=2.80, p=0.04). Additionally, 24h-MBs compositions were significantly associated with WC when using ENMO or MAD (F=3.11, p=0.03; F=2.75, p=0.04, respectively). All 24h-MBs compositions, except for CPM VA, were significantly associated with fat% (ENMO F=3.20, p=0.02; MAD F=2.68, p=0.05; CPM VM F=3.27, p=0.02) [See S7Table 1].

Within the time reallocation models, the direction of association between 24h-MBs composition and significantly associated cardiometabolic variables (BMI, WC, Fat% and HbA1c) differed across metrics [see S7 Fig. 1]. ENMO showed significant results for BMI and HbA1c, where reallocating 20 minutes into MVPA proportionally from the remaining behaviors was associated with a decrease of -0.8 kg/m² in BMI ([-1.3;-0.25], 2.9% decrease, ES=-0.15) and a decrease in HbA1c of -0.58 mmol/l ([-1.02;-0.14], 1.58% decrease, ES=-0.09). However, reallocating time to more LPA was associated with an increase in HbA1c of +0.85 mmol/l ([0.19; 1.5], 2.32% increase, ES=0.13). For WC, ENMO and MAD had similar results for reallocating time into more MVPA (+20 minutes), with a decrease of -1.8 cm in WC ([-3.24;-0.35], 1.9% decrease, ES=-0.12) for ENMO and -1.13 cm ([-2.13;-0.12], 1.19% decrease, ES=-0.08) for MAD. Moreover, reallocating time out of sleep and proportionally to the other behaviors was associated with a significant decrease in WC of -0.6 cm ([-1.03;-0.08], 0.63% decrease, ES=-0.04) regarding the ENMO metric only. For fat%, ENMO, MAD and CPM VM showed similar favorable associations for reallocating time out of sleep. However, the estimated differences in fat% were small (ENMO -0.42% [-0.73;-0.11], 1.33% decrease, ES=-0.05; MAD -0.35% [-0.65;-0.05], 1.11% decrease, ES=-0.04; and CPM VM -0.32% [-0.62;-0.03], 1.01% decrease, ES=-0.03). Improvements in fat% were observed for ENMO when reallocating time into MVPA (-1.02% [-1.93;-0.09], 3.22% decrease, ES=-1.11) and into LPA for CPM VM (-0.41% [-0.69;-0.14], -1.30% decrease, ES=-0.04). Nevertheless, unfavorable small changes were observed for fat% when reallocating time into more SB for ENMO (-0.37% [-0.69;-0.04], -1.17% decrease, ES=-0.04).

**Cut-point independent movement behaviors features**

For average acceleration, only MAD had a significant negative association with BMI (t=-2.52, p=0.01) and fat% (t=-2.03, p=0.04), and it had a positive association with WC (t=3.68, p<0.001). The intensity gradient was negatively associated with BMI for ENMO (t=-1.98, p=0.05), MAD (t=-2.72, p<0.01) and CMP VA (t=-2.48, p=0.01), as well as with WC for MAD (t=-2.27, p=0.02). However, the intensity gradient was positively associated with BMI (t=2.53, p=0.01) and WC (t=2.5, p=0.01) for CPM VM. [See S7 Table 2].

| **24h-MBs composition** | | | | | | | | | | | | | | | | |
| --- | --- | --- | --- | --- | --- | --- | --- | --- | --- | --- | --- | --- | --- | --- | --- | --- |
|  | ENMO | | | | MAD | | | | CPM VA | | | | CPM VM | | | |
|  | Sum Sq | F | p | Adj. R² | Sum Sq | F | p | Adj. R² | Sum Sq | F | p | Adj. R² | Sum Sq | F | p | Adj. R² |
| BMI | 0.300 | 3.230 | **0.020** | 0.160 | 0.226 | 2.439 | *0.070* | 0.150 | 0.166 | 1.776 | 0.153 | 0.140 | 0.300 | 3.300 | 0.200 | 0.170 |
| WC | 0.164 | 3.106 | **0.030** | 0.270 | 0.150 | 2.750 | **0.040** | 0.270 | 0.105 | 1.965 | 0.121 | 0.260 | 0.180 | 3.420 | 0.200 | 0.270 |
| WHR | 0.004 | 0.261 | 0.853 | 0.360 | 0.001 | 0.034 | 0.410 | 0.360 | 0.002 | 0.127 | 0.940 | 0.360 | 0.010 | 0.830 | 0.470 | 0.360 |
| Glucose | 0.131 | 2.222 | *0.087* | 0.450 | 0.022 | 0.360 | 0.780 | 0.440 | 0.070 | 1.248 | 0.293 | 0.450 | 0.050 | 0.880 | 0.450 | 0.440 |
| HbA1c | 0.088 | 2.798 | **0.042** | 0.540 | 0.027 | 0.813 | 0.490 | 0.530 | 0.030 | 1.000 | 0.390 | 0.530 | 0.003 | 0.090 | 0.960 | 0.520 |
| HDL-Cholesterol | 0.033 | 0.296 | 0.828 | 0.250 | 0.047 | 0.424 | 0.740 | 0.250 | 0.068 | 0.614 | 0.607 | 0.250 | 0.210 | 1.890 | 0.130 | 0.260 |
| LDL-Cholesterol | 0.299 | 1.474 | 0.223 | 0.140 | 0.306 | 1.513 | 0.210 | 0.140 | 0.357 | 1.769 | 0.154 | 0.150 | 0.370 | 1.840 | 0.140 | 0.150 |
| Total Cholesterol | 0.136 | 1.546 | 0.200 | 0.140 | 0.130 | 1.476 | 0.220 | 0.130 | 0.160 | 1.827 | 0.144 | 0.140 | 0.200 | 2.270 | *0.080* | 0.150 |
| Triglycerides | 0.022 | 0.035 | 0.991 | 0.130 | 0.025 | 0.039 | 0.990 | 0.130 | 0.050 | 0.078 | 0.970 | 0.130 | 0.297 | 0.460 | 0.710 | 0.140 |
| Fat% | 0.472 | 3.202 | **0.020** | 0.470 | 0.400 | 2.680 | **0.050** | 0.470 | 0.340 | 2.270 | *0.080* | 0.470 | 0.480 | 3.270 | **0.020** | 0.470 |
| Systolic BP | 0.015 | 0.403 | 0.750 | 0.180 | 0.007 | 0.198 | 0.920 | 0.180 | 0.011 | 0.298 | 0.826 | 0.180 | 0.040 | 1.080 | 0.360 | 0.190 |
| Diastolic BP | 0.042 | 1.150 | 0.330 | 0.120 | 0.046 | 1.244 | 0.300 | 0.130 | 0.038 | 1.055 | 0.369 | 0.120 | 0.050 | 1.470 | 0.220 | 0.130 |

S7 Table 1. Associations between 24-hour movement behavior compositions and cardiometabolic health parameters regarding the four metrics (ENMO, MAD, CPM VA, CPM VM)

Footnote. BMI: body mass index, WC: waist circumference, WHR: waist-to-hip ratio, BP: blood pressure, Sum sq: sum square, and Numerator degrees of freedom= 3 for all F tests. All models were adjusted for sex, age, education level, smoking status and medication intake and pathology. All variables were log-transformed and the bold values indicate p<0.05. The intensity-based cut-points thresholds for each metric are as follows: ENMO Hildebrand et al. (2014), MAD Vaha Ypya et al. (2018, 2023), CPM VA Troiano et al. (2008), and CPM VM Sasaki et al. (2011).

S7 Fig. 1 Estimated changes in cardiometabolic parameters associated with time reallocations into one behavior proportionally collected out of the other behaviors.


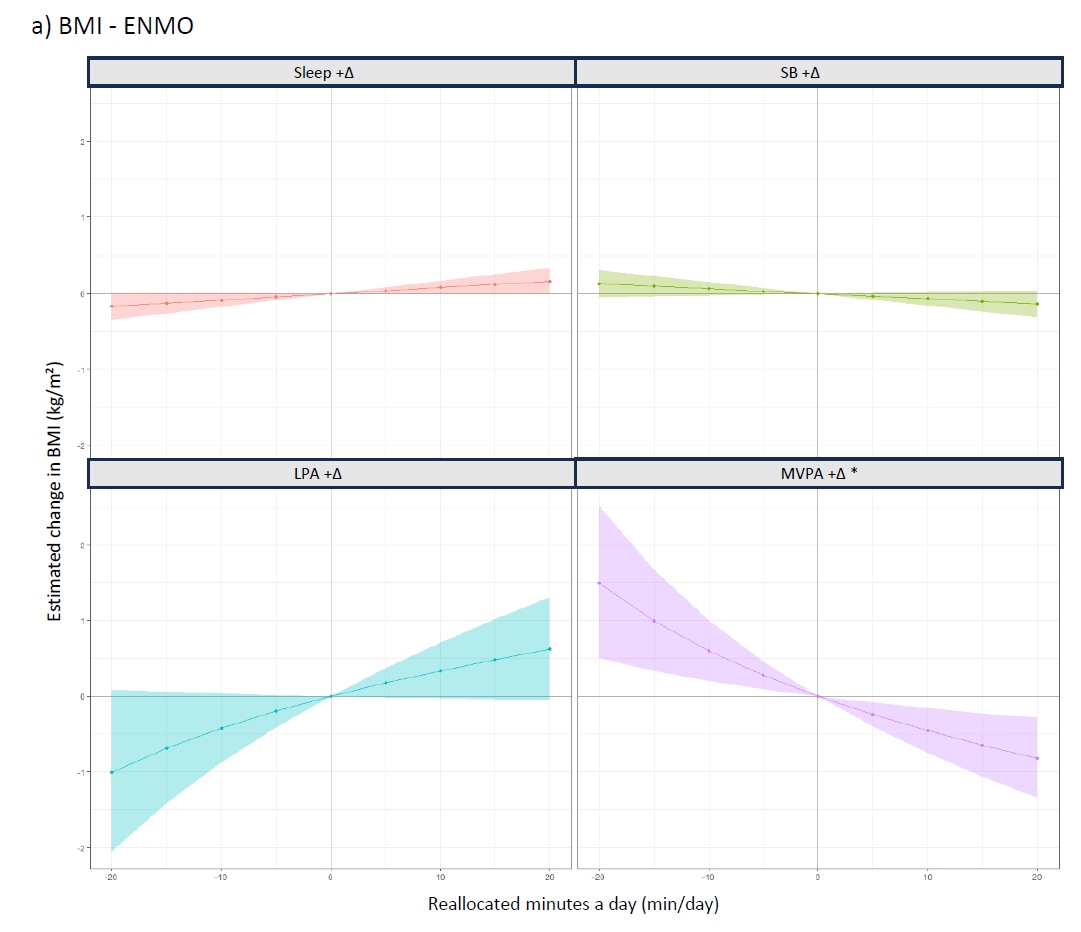


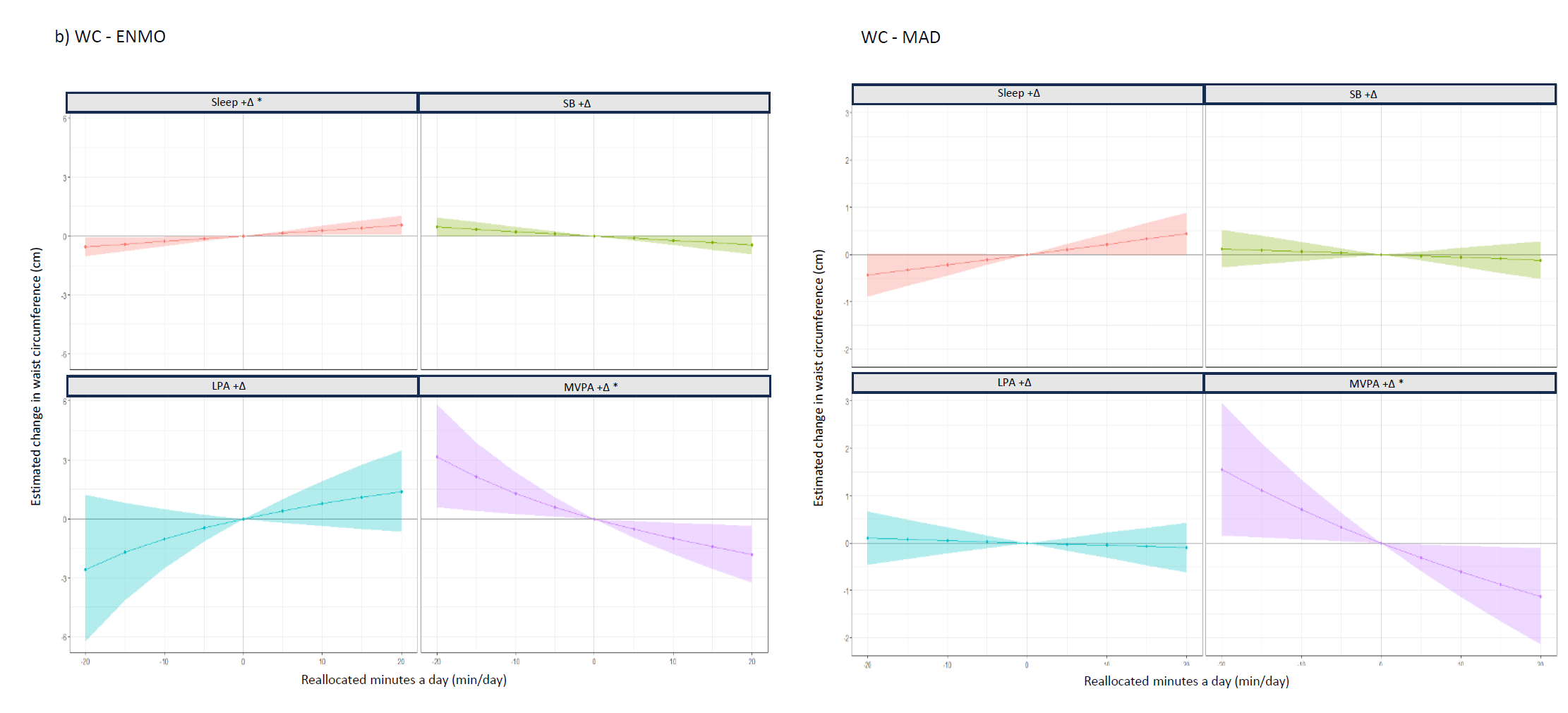


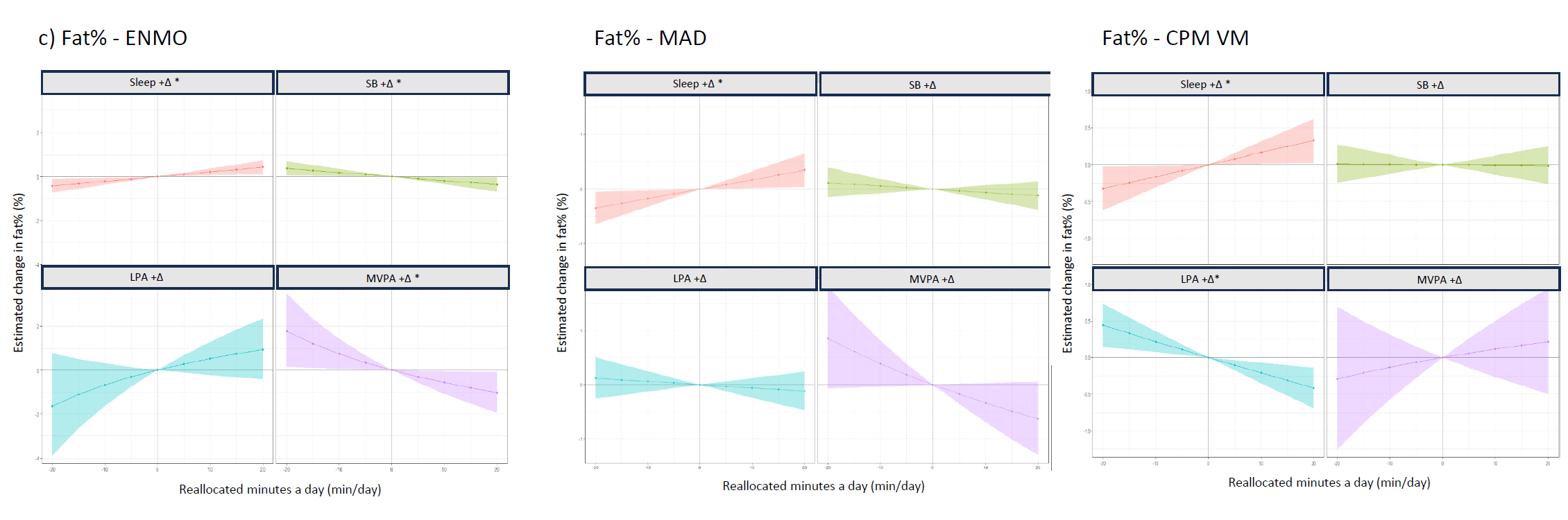


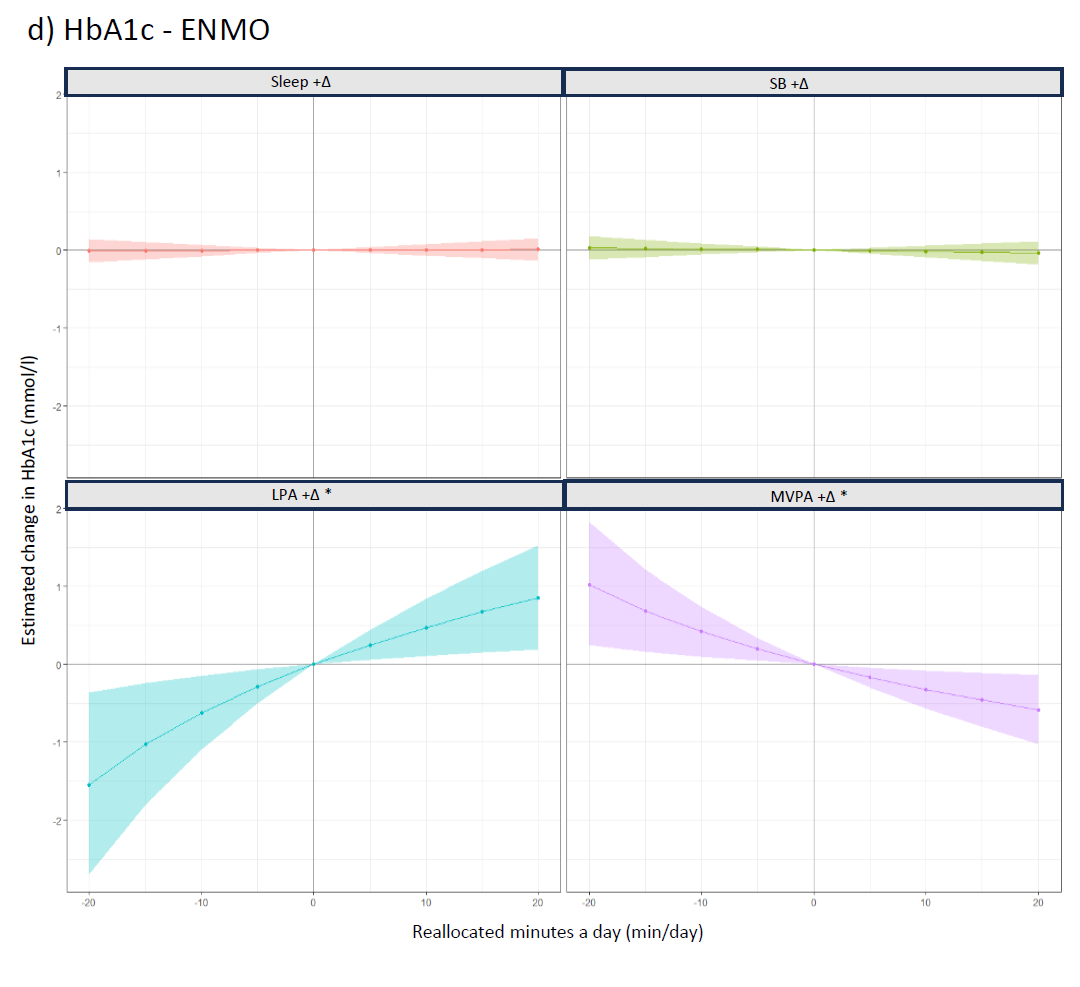
Body mass index (BMI), b) waist circumference, c) fat%, and d) HbA1c. For example, red lines represent reallocations of time ranging from -20 minutes to +20 minutes into sleep (sleep +Δ) out of sedentary behavior (SB-Δ), light physical activity (LPA -Δ), or moderate to vigorous physical activity (MVPA -Δ). SB: sedentary behavior, LPA: light physical activity, MVPA: moderate to vigorous physical activity. The X-axis represents the minutes a day (min/day) reallocated, ranging from -20 minutes to +20 minutes. The Y-axis represents the predicted difference in outcome for BMI in kg/m², waist circumference in cm, fat percentage in % and HbA1c in mmol/l. All models were adjusted for sex, age, education level, smoking status, medication intake and pathology, and all log-transformed variables were back-transformed to enhance interpretability. * indicates significant time reallocation. The intensity-based cut-points thresholds for each metric are as follows: ENMO Hildebrand et al. (2014), MAD Vaha Ypya et al. (2018, 2023), CPM VA Troiano et al. (2008), and CPM VM Sasaki et al. (2011).

| **Intensity gradient** | | | | | | | | | | | | | | | | | | | | |
| --- | --- | --- | --- | --- | --- | --- | --- | --- | --- | --- | --- | --- | --- | --- | --- | --- | --- | --- | --- | --- |
|  | ENMO | | | | | MAD | | | | | CPM VA | | | | | CPM VM | | | | |
|  | est | st error | t | p | Adj. R² | est | st error | t | p | Adj. R² | est | st error | t | p | Adj. R² | est | st error | t | p | Adj. R² |
| BMI | -0.092 | 0.046 | -1.984 | **0.049** | 0.140 | -0.166 | 0.061 | -2.718 | **0.007** | 0.154 | -0.170 | 0.069 | -2.475 | **0.014** | 0.149 | 0.260 | 0.103 | 2.527 | **0.012** | 0.150 |
| WC | -0.052 | 0.035 | -1.459 | 0.146 | 0.250 | -0.105 | 0.046 | -2.270 | **0.024** | 0.261 | -0.095 | 0.053 | -1.802 | *0.073* | 0.254 | 0.195 | 0.078 | 2.497 | **0.013** | 0.265 |
| WHR | 0.002 | 0.020 | 0.125 | 0.901 | 0.364 | -0.013 | 0.026 | -0.504 | 0.615 | 0.365 | 0.010 | 0.029 | 0.342 | 0.733 | 0.365 | 0.063 | 0.044 | 1.437 | 0.152 | 0.371 |
| Glucose | 0.018 | 0.038 | 0.464 | 0.643 | 0.441 | 0.009 | 0.050 | 0.182 | 0.856 | 0.440 | -0.025 | 0.056 | -0.436 | 0.663 | 0.441 | 0.048 | 0.084 | 0.566 | 0.572 | 0.441 |
| HbA1c | -0.039 | 0.028 | -1.381 | 0.169 | 0.534 | -0.066 | 0.036 | -1.819 | *0.071* | 0.537 | -0.074 | 0.041 | -1.808 | *0.072* | 0.537 | -0.016 | 0.063 | -0.247 | 0.805 | 0.529 |
| HDL-Cholesterol | 0.034 | 0.051 | 0.660 | 0.510 | 0.252 | 0.050 | 0.067 | 0.750 | 0.454 | 0.253 | 0.022 | 0.076 | 0.288 | 0.774 | 0.251 | -0.113 | 0.113 | -0.999 | 0.319 | 0.254 |
| LDL-Cholesterol | -0.062 | 0.069 | -0.902 | 0.368 | 0.137 | -0.048 | 0.091 | -0.524 | 0.601 | 0.135 | -0.113 | 0.103 | -1.102 | 0.272 | 0.139 | -0.129 | 0.154 | -0.843 | 0.400 | 0.137 |
| Total Cholesterol | -0.038 | 0.046 | -0.840 | 0.402 | 0.127 | -0.030 | 0.060 | -0.505 | 0.614 | 0.125 | -0.084 | 0.068 | -1.250 | 0.213 | 0.130 | -0.119 | 0.101 | -1.181 | 0.239 | 0.130 |
| Triglycerides | -0.003 | 0.122 | -0.022 | 0.982 | 0.140 | -0.063 | 0.161 | -0.391 | 0.696 | 0.140 | -0.065 | 0.181 | -0.358 | 0.721 | 0.140 | 0.193 | 0.271 | 0.712 | 0.477 | 0.142 |
| Fat% | -0.072 | 0.064 | -1.130 | 0.260 | 0.455 | -0.152 | 0.085 | -1.774 | *0.078* | 0.461 | -0.142 | 0.095 | -1.499 | 0.136 | 0.458 | 0.299 | 0.140 | 2.129 | **0.035** | 0.465 |
| Systolic BP | 0.003 | 0.029 | 0.109 | 0.913 | 0.182 | -0.005 | 0.039 | -0.134 | 0.893 | 0.182 | 0.019 | 0.044 | 0.429 | 0.668 | 0.183 | 0.121 | 0.065 | 1.848 | *0.066* | 0.196 |
| Diastolic BP | -0.020 | 0.029 | -0.672 | 0.502 | 0.121 | -0.041 | 0.038 | -1.064 | 0.289 | 0.124 | 0.002 | 0.044 | 0.039 | 0.969 | 0.119 | 0.107 | 0.065 | 1.654 | 0.100 | 0.131 |

S7 Table 2. Associations between intensity gradient and average acceleration and cardiometabolic health variables according to the four metrics (ENMO, MAD, CPM VA, and CPM VM)

| **Average acceleration** | | | | | | | | | | | | | | | | | | | | |
| --- | --- | --- | --- | --- | --- | --- | --- | --- | --- | --- | --- | --- | --- | --- | --- | --- | --- | --- | --- | --- |
|  | ENMO | | | | | MAD | | | | | CPM VA | | | | | CPM VM | | | | |
|  | est | st error | t | p | adj R² | est | st error | t | p | adj R² | est | st error | t | p | adj R² | est | st error | t | p | adj R² |
| BMI | -0.004 | 0.002 | -1.661 | *0.098* | 0.135 | -0.004 | 0.001 | -2.517 | **0.013** | 0.150 | -0.002 | 0.001 | -1.428 | 0.155 | 0.132 | 0.000 | 0.001 | -0.618 | 0.537 | 0.124 |
| WC | -0.002 | 0.002 | -1.420 | 0.157 | 0.250 | 0.077 | 0.021 | 3.678 | **0.000** | 0.261 | -0.001 | 0.001 | -1.254 | 0.211 | 0.248 | 0.000 | 0.001 | -0.408 | 0.684 | 0.243 |
| WHR | 0.000 | 0.001 | 0.370 | 0.712 | 0.365 | 0.000 | 0.001 | -0.235 | 0.814 | 0.364 | 0.000 | 0.001 | 0.678 | 0.499 | 0.366 | 0.000 | 0.000 | 1.040 | 0.299 | 0.368 |
| Glucose | 0.002 | 0.002 | 1.012 | 0.313 | 0.443 | 0.000 | 0.001 | 0.301 | 0.764 | 0.441 | 0.000 | 0.001 | 0.351 | 0.726 | 0.441 | 0.001 | 0.001 | 1.409 | 0.160 | 0.446 |
| HbA1c | -0.001 | 0.001 | -1.041 | 0.299 | 0.531 | -0.001 | 0.001 | -1.189 | 0.236 | 0.532 | -0.001 | 0.001 | -0.723 | 0.470 | 0.530 | 0.000 | 0.000 | -0.026 | 0.979 | 0.529 |
| HDL-Cholesterol | 0.003 | 0.002 | 1.342 | 0.181 | 0.257 | 0.002 | 0.002 | 1.375 | 0.171 | 0.258 | 0.001 | 0.001 | 1.092 | 0.276 | 0.255 | 0.001 | 0.001 | 0.801 | 0.424 | 0.253 |
| LDL-Cholesterol | -0.002 | 0.003 | -0.681 | 0.497 | 0.136 | -0.001 | 0.002 | -0.276 | 0.783 | 0.134 | -0.001 | 0.002 | -0.811 | 0.418 | 0.137 | -0.001 | 0.001 | -0.827 | 0.409 | 0.137 |
| Total Cholesterol | -0.001 | 0.002 | -0.622 | 0.534 | 0.125 | 0.000 | 0.001 | -0.218 | 0.828 | 0.124 | -0.001 | 0.001 | -0.930 | 0.354 | 0.127 | -0.001 | 0.001 | -0.999 | 0.319 | 0.128 |
| Triglycerides | 0.000 | 0.006 | 0.035 | 0.972 | 0.140 | -0.001 | 0.004 | -0.170 | 0.865 | 0.140 | 0.000 | 0.003 | -0.050 | 0.960 | 0.140 | 0.001 | 0.002 | 0.284 | 0.777 | 0.140 |
| Fat% | -0.004 | 0.003 | -1.308 | 0.193 | 0.457 | -0.004 | 0.002 | -2.027 | **0.044** | 0.464 | -0.002 | 0.002 | -1.296 | 0.197 | 0.457 | -0.001 | 0.001 | -0.859 | 0.392 | 0.454 |
| Systolic BP | 0.001 | 0.001 | 0.907 | 0.365 | 0.185 | 0.000 | 0.001 | 0.506 | 0.613 | 0.183 | 0.000 | 0.001 | 0.315 | 0.753 | 0.182 | 0.000 | 0.000 | 0.829 | 0.408 | 0.185 |
| Diastolic BP | 0.000 | 0.001 | -0.054 | 0.957 | 0.119 | 0.000 | 0.001 | -0.552 | 0.582 | 0.120 | 0.000 | 0.001 | -0.460 | 0.646 | 0.120 | 0.000 | 0.000 | -0.195 | 0.846 | 0.119 |

Footnote. BMI: body mass index, WC: waist circumference, WHR: waist-to-hip ratio, BP: blood pressure, and number of degrees of freedom= 3 for all F tests. All the models were adjusted for sex, age, education level, smoking status, medication intake and pathology. All the variables were log-transformed, and the bold values indicate p<0.05. The intensity-based cut- points thresholds for each metric are as follows: ENMO Hildebrand et al. (2014), MAD Vaha Ypya et al. (2018, 2023), CPM VA Troiano et al. (2008), and CPM VM Sasaki et al. (2011).
