# Supplementary material for: A comparative analysis of 24-hour movement behaviors features using different accelerometer metrics in adults: Implications for guideline compliance and associations with cardiometabolic health
Source: PLoS One. 2024 Sep 17;19(9):e0309931. doi: 10.1371/journal.pone.0309931 (PMC11407674; doi:10.1371/journal.pone.0309931)
Supplement: Attachment — Submitted filename: Response to reviewers.docx [file pone.0309931.s009.docx]

We would like to thank the editors and the reviewers for carefully reviewing our manuscript and the appreciation of our work. We believe the constructive comments and suggestions improved the quality of the manuscript. All changes are visible with track changes in the manuscript and are also integrated in this rebuttal letter/point-by-point reply.

# Reviewer #1:

The results indicate that the movement behavior data varied depending on the metric used for analysis. Specifically, ENMO (Euclidean Norm Minus One) represented the most sedentary movement behavior profile, while CPM (Counts Per Minute) vector magnitude represented the most active profile.
This suggests that different accelerometer metrics capture different aspects of movement behavior, with some metrics highlighting more sedentary patterns while others emphasize more active behaviors. Understanding these differences is crucial for accurately assessing individuals' activity levels and sedentary behavior, which in turn can inform interventions aimed at promoting physical activity and reducing sedentary time to improve overall health outcomes.
Interestingly, the study found that reallocating time towards moderate-to-vigorous physical activity consistently predicted significant improvements in cardiometabolic variables, with the exception of fat percentage. This suggests that increasing time spent in moderate-to-vigorous physical activity may have positive effects on various aspects of cardiometabolic health, highlighting the importance of engaging in activities that elevate heart rate and promote greater exertion.
Overall, these findings emphasize the complexity of studying movement behaviors and their associations with health outcomes, indicating that the choice of accelerometer metric can influence both compliance rates with guidelines and the observed relationships with cardiometabolic variables.
Overall, the study suggests variations in agreement between different metrics, with some showing better consistency than others across various activity intensities. These findings underscore the importance of considering the choice of metric carefully when analyzing accelerometer data for physical activity assessment. Average acceleration showed strong associations with MVPA across all metrics, its relationship with LPA was weaker. Additionally, the intensity gradient exhibited stronger correlations with MVPA compared to LPA, with some variation across metrics.
The choice of metric significantly influences the composition of 24h MBs, particularly in terms of the distribution of time spent in sedentary behavior, light physical activity, and moderate to vigorous physical activity. ENMO tended to allocate more time to sedentary behavior, while CPM VM favored light physical activity, and MAD had a higher proportion of moderate to vigorous physical activity.
The analysis revealed significant associations between time spent on 24h movement behaviors (MB) and various cardiometabolic variables, with differences observed across different accelerometer metrics. Overall, these findings underscore the importance of considering different accelerometer metrics when examining associations between physical activity behaviors and cardiometabolic health outcomes. The direction and magnitude of associations varied across metrics, highlighting the need for tailored interventions aimed at promoting specific types of physical activity to improve cardiometabolic health.

The study has several limitations that should be considered when interpreting the results:

**1. Selection of Cutoff Points**: The use of cutoff points for each accelerometer metric is a potential limitation. While commonly used cutoff points were selected, there are alternative cutoff points available. These cutoff points are typically based on specific validation protocols conducted in particular populations. Therefore, the choice of cutoff points may impact the interpretation of physical activity data and comparisons across studies.

**2. Limited Generalizability:** The study only used hip-worn accelerometer data for the waking day. This may limit the generalizability of the findings, particularly when comparing them to studies using wrist-worn accelerometer data. Differences in acceleration patterns based on body location can influence the assessment of physical activity levels. Therefore, caution is needed when generalizing findings to populations or studies using different accelerometer placements.

**3. Sample Characteristics:** The results may be influenced by the characteristics of the sample population studied. Demographic factors such as age, gender, and physical fitness levels can affect physical activity patterns and associations with health outcomes. Therefore, the findings may not be representative of other populations with different demographic profiles.

**4. Cross-Sectional Design:** The study likely employed a cross-sectional design, which limits the ability to establish causal relationships between physical activity behaviors and cardiometabolic health outcomes. Longitudinal studies are needed to better understand the temporal relationships between these variables and to assess the effectiveness of interventions.

**5. Measurement Error:** Accelerometer measurements are subject to measurement error, which can arise from factors such as device malfunction, wear time compliance, and data processing methods. These errors could potentially affect the accuracy and reliability of the physical activity measurements and subsequent associations with health outcomes.

#### Acknowledging these limitations can help researchers and clinicians better interpret the study findings and guide future research efforts aimed at addressing these limitations to improve the understanding of physical activity and its impact on health.

**Anwser:** Thank you for the summary of the paper. We agree that the limitations mentioned above can improve interpretability of the study findings. Most of the limitations that were mentioned were already addressed in this paper. Therefore, we have added some sentences for additional clarification where necessary.

1. Selection of cut off points: The limitations linked to the selection of cut-points are already clearly mentioned in the discussion. However, we added an additional sentence to make this more clear.

**Changes in manuscript**

Discussion line 477-479: Although commonly used cut-points were selected, other cut-points are available, which are all based on a specific validation protocol in a specific sample. Using other cut-points might provide other results.

2. Limited generalizability: This limitation was already mentioned in the discussion line 485 – 487. ***“Finally, only hip-worn data for the waking day were used in this study, which hinders comparison with studies using wrist-worn data due to differences in acceleration based on body location.”***
3. Sample characteristics: Since the aim of this study was to compare metrics on the same dataset, the sample characteristics are subordinate to the research question. It is our opinion that it is less important to mention the sample characteristics as a limitation. Therefore, we have chosen not to include this in the discussion/limitations section.
4. Cross-sectional design: Associations between time-use and cardiovascular health parameters were moved to the Supporting Information S7_docx. Within the supporting information, we have added one sentence to highlight the inability to infer causal relationships.

**Changes in manuscript**

Supporting Information S7_docx: The choice of data processing metric has an impact on the time spent in movement behavior features, average acceleration and intensity gradient. This supplementary information shows the impact of different 24h-MBs compositions, average acceleration and intensity gradient on the association with cardiometabolic health variables. However, because the data are cross-sectional causality cannot be inferred.

5. Measurement error: Devices with high calibration errors (n=1) were excluded. To ensure comparability, all datafiles were analysed with the same GGIR script, only deviating for the metrics used. See method line 147-149. ***“The GGIR package uses an autocalibration algorithm that checks and corrects for calibration errors in triaxial accelerometer signals [12]. Actigraph files (n=1) with a postcalibration error greater than 0.01 g were excluded [12].”*** This was also mentioned in the discussion line 474-476. ***“******Perfect reproducibility across the four metrics was ensured by the use of the GGIR package, which allows for consistent data reduction features, i.e., autocalibration, sleep algorithm, and nonwear detection methods.”***

# Reviewer #2:

This paper focuses on the different metrics available in the GGIR package for accelerometer processing. This is quite a technical aspect of physical activity measures, but an important one, especially in light of the findings which show that the different metrics can produce quite different summary estimates. In general, I think this is a good, well-written, useful paper. However there are areas which are unclear and I feel it currently tries to tackle too much. The discussion would also benefit from less focus on just repeating the results and more on what this means for someone about to begin a study – which metric should they use and why? I list some more detailed points below.

Major points

#### 1. Currently, definitions and advantages and disadvantages of the different processing metrics and PA summary measures don’t appear until the methods section (eg ENMO at line 142, intensity gradient line 160+), but this makes it very hard to understand the background and why these different metrics and summaries (and hence the manuscript itself) are important. As this manuscript involves very technical aspects of accelerometer processing which not all readers will be familiar with, I suggest adding a clear overview of the full process (raw data to processed data to cut points & summaries) early on in the background. Then describe the different processing metrics and the different PA measures - definitions, where they fit into this process, how they differ and how it might be expected to affect the summaries.

**Answer:** We agree and, as suggested, we now provide additional clarification in the background section. We now commence with a description of tri-axial accelerometers as the preferred method for analyzing 24-hour movement behaviors, highlighting the transition from processing 'counts per minute' data with closed-source software specific tools to processing raw accelerometer data with open-source tools. Next, we outline the derived outcomes from these data, including time spent in movement behaviors based on cut-points for classifying activity intensities, alongside newer features like cut-point-independent average acceleration and intensity gradient. We then proceed with the explanation of the concept of metrics, accompanied by cut-points for categorizing behaviors into activity intensities. Commonly used metrics such as ENMO, MAD, CPM VA, and CPM VM are highlighted, with the recognition that no gold standard exists for the selection of one metric.

To enhance clarity, we have supplemented the background section with additional explanations in Table 1. This table now contains more detailed information (definition, formula and GGIR specifications) on the various metrics.

**Changes in manuscript:**

Background lines 42-90: To better understand 24h-MBs in adults, it is crucial to accurately measure these behaviors by measurement tools such as tri-axial accelerometers (e.g., the Actigraph GT3X+) [6]. These measurement tools are the preferred method for collecting 24h-MBs as they quantify accelerations in orthogonal directions of a three dimensional space [6,7]. There has been a shift from analyzing accelerometer data using "activity counts per minute" generated by closed-source proprietary accelerometer brand-specific algorithms (e.g. ActiLife software for Actigraph accelerometers) toward analyzing raw gravitational acceleration data (m/s²) [8]. Raw acceleration data allows for open-source data processing, such as the R package GGIR, which can be used regardless of the type of accelerometer [8,9]. Output from this open-source raw data package can be classified as time spent in movement behaviors defined by cut-points to classify activity intensities, as well as newer cut-point independent movement behavior features such as average acceleration and intensity distribution of activity throughout a day. These cut-point independent movement behavior features enhance the comparability between studies [10,11].

Nevertheless, working with raw data still requires the use of data reduction methods, also called metrics, to separate the acceleration signal from the gravitation signal [8,9]. Different metrics exist and these can be distinguished based on the method for extracting the acceleration signal [7,8]. Commonly used data reduction metrics for processing raw accelerometer data in GGIRare the Euclidian Norm Minus One (ENMO) and Mean Amplitude Deviation (MAD) as these analytic techniques are perceived as not too complex for users and they have the ability of quantifying output in universal units instead of abstract scales [see Supporting Information S1 for more details] [8,9,12]. As the shift from using activity counts cut-points to classify activity intensities into raw accelerometer data processing is still evolving, a new metric that replicates the closed-source Actilife software was developed in the GGIR package, i.e., the “counts per minute” (CPM) metric [13]. This CPM metric has the ability to process data of the vertical axis (VA) only or to work with the vector magnitude (VM) [See S1] [13]. The main advantage of this new metric in GGIR is the reduction of human errors. Data processing in ActiLife software requires manual processing of data to define wear and nonwear times, and the GGIR package applies the same nonwear algorithm on each data file [13]. Despite the popularity of working with accelerometer data, no gold standard exist for the most appropriate activity intensity-based cut-point accompanied by a data reduction metric. This lack of standardization affects the time spent in movement behavior and hampers comparability between studies [14].

Previous studies have highlighted that there are differences in cut-point dependent PA and SB durations depending on whether they are derived from raw accelerometer data or “counts per minute” data [14,15]. In contrast, literature shows comparable findings for cut-point independent average acceleration and intensity gradient across the acceleration metrics ENMO and MAD [16]. Interestingly, although 24h-MBs are codependent, none of these studies interpreting time spent engaged in behaviors used a compositional behavioral approach but focused on one or more behaviors in isolation (e.g., PA, SB). Moreover, no previous studies have compared three different movement behavior features (i.e., time spent in a 24h period, overall activity volume, and overall activity intensity) between the new CPM metric for VA and VM, the ENMO metric and the MAD metric with hip-worn accelerometer data in adults.

Supporting information S1_Table: See S1_table for adjustments.

#### 2. This manuscript is trying to do a lot of things, which makes it difficult to follow. In particular, associations between PA measures and cardiovascular outcomes seem out of place – they’re not affected directly by the processing metrics, the analysis is not in-depth enough to be a full association study and its not clear what the implications are for different metrics. I think the paper would be stronger and clearer if this section was dropped entirely. If the authors do decide to keep this, then there needs to be more linking to the metrics and crucially some guidance on the appropriate metrics to use in different circumstances.

**Answer:** We agree, and have decided to move the analysis of associations between 24h compositions and cardiovascular health into a supplementary file. We do believe that this supplementary information can create awareness for time-use researchers about the impact of metric use on study results when interpreting and comparing data.

**Changes in manuscript**

Methods line 228-229: The indepth analysis of these models are reported in the Supporting Information S7.

Results line 335-387: The time spent on 24h-MB was significantly associated with BMI and HbA1c when ENMO was used (BMI: F= 3.23, p=0.02; HbA1c: F=2.80, p=0.04). Additionally, 24h-MB compositions were significantly associated with WC when using ENMO or MAD (F=3.11, p=0.03; F=2.75, p=0.04, respectively). All 24h-MB compositions, except for CPM VA, were significantly associated with fat% (ENMO F=3.20, p=0.02; MAD F=2.68, p=0.05; CPM VM F=3.27, p=0.02). For average acceleration, only MAD had a significant negative association with BMI (t=-2.52, p=0.01) and fat% (t=-2.03, p=0.04), and it had a positive association with WC (t=3.68, p<0.001). The intensity gradient was negatively associated with BMI for ENMO (t=-1.98, p=0.05), MAD (t=-2.72, p<0.01) and CMP VA (t=-2.48, p=0.01), as well as with WC for MAD (t=-2.27, p=0.02). However, the intensity gradient was positively associated with BMI (t=2.53, p=0.01) and WC (t=2.5, p=0.01) for CPM VM. See supporting Information S7 for more in depth analysis of these linear models.

~~Within the time reallocation models, the direction of association between 24h-MB composition and significantly associated cardiometabolic variables (BMI, WC, Fat% and HbA1c) differed across metrics [see Fig. 1]. ENMO showed significant results for BMI and HbA1c, where reallocating 20 minutes into MVPA proportionally from the remaining behaviors was associated with a decrease of -0.8 kg/m² in BMI ([-1.3;-0.25], 2.9% decrease, ES=-0.15) and a decrease in HbA1c of -0.58 mmol/l ([-1.02;-0.14], 1.58% decrease, ES=-0.09). However, reallocating time to more LPA was associated with an increase in HbA1c of +0.85 mmol/l ([0.19; 1.5], 2.32% increase, ES=0.13). For WC, ENMO and MAD had similar results for reallocating time into more MVPA (+20 minutes), with a decrease of -1.8 cm in WC ([-3.24;-0.35], 1.9% decrease, ES=-0.12) for ENMO and -1.13 cm ([-2.13;-0.12], 1.19% decrease, ES=-0.08) for MAD. Moreover, reallocating time out of sleep and proportionally to the other behaviors was associated with a significant decrease in WC of -0.6 cm ([-1.03;-0.08], 0.63% decrease, ES=-0.04) regarding the ENMO metric only. For fat%, ENMO, MAD and CPM VM showed similar favorable associations for reallocating time out of sleep. However, the estimated differences in fat% were small (ENMO -0.42% [-0.73;-0.11], 1.33% decrease, ES=-0.05; MAD -0.35% [-0.65;-0.05], 1.11% decrease, ES=-0.04; and CPM VM -0.32% [-0.62;-0.03], 1.01% decrease, ES=-0.03). Improvements in fat% were observed for ENMO when reallocating time into MVPA (-1.02% [-1.93;-0.09], 3.22% decrease, ES=-1.11) and into LPA for CPM VM (-0.41% [-0.69;-0.14], -1.30% decrease, ES=-0.04). Nevertheless, unfavorable small changes were observed for fat% when reallocating time into more SB for ENMO (-0.37% [-0.69;-0.04], -1.17% decrease, ES=-0.04).~~

Supporting information:

S7. Docx. Comparison of associations between movement behavior features and cardiometabolic variables.
The choice of data processing metric has an impact on the time spent in movement behavior features, average acceleration and intensity gradient. This supplementary information shows the impact of data processing metric use on the association between different 24h-MBs compositions, average acceleration and intensity gradient and cardiometabolic health variables. However, because the data are cross-sectional causality cannot be inferred.

Please see amended supplementary file S7 for the results, tables and figure which were originally in the manuscript but are now embedded in one supplementary file.

#### 3. Much of the model description in the methods is not clear, so I am unable to comment on the suitability of the modelling. For example, at line 192 – what’s the outcome in this model? What fixed effect terms are included? What are the random terms? Line 192 refers to a single model, but line 194 to multiple models –how many models and what are they? Also, line 193 refers to random slopes, but line 194 talks about random intercept models (ie random intercept only) – which is it? Finally, I think there might be repeated measures on the same individual in here – is there a random intercept for participant included? A model equation would be useful, eg in the Supporting Information.

**Answer:** In response we have added clarification in the methods section. Moreover, we refer to a paper of Lim et al. 2023 which includes the R-code we used to set up our model.

**Changes in manuscript**

Methods line 201-214: Compositional Data Analysis (CoDA) was used to account for the codependency of the 24h-MBs using the R packages *compositions* and *codaredistlm* [26,27]. The 24h-MB compositions created by each accelerometer metric (consisting of sleep, SB, LPA, and MVPA) were expressed as four sets of three isometric log-ratios (ILRs) [28]. Variation matrices were created to explore the variance covariance of the 24h-MBs [see Supporting Information S3] [28]. Linear mixed effects models were used to explore differences between compositions. The dependent variables were the ILRs, in long ‘stacked’ format with a dummy variable indicating whether they were ILR1, ILR2 or ILR3. As described in Lim and colleagues [29], random slopes were added at the log ratio level, grouped by participant ID, and repeated within participant ID (random intercept) for each accelerometer metric composition. Fixed effect interactions between the metrics and the dummy variable representing the ILR number were analyzed to test for differences between the different accelerometer metrics (MANOVA F-test). Full models included additional interactions to adjust for covariates.

References: 29. Lim R, Dumuid D, Parfitt G, Stanford T, Post D, Bilton R, et al. Using wrist-worn accelerometers to identify the impact of medicines with anticholinergic or sedative properties on sedentary time: A 12-month prospective analysis. Maturitas. 2023;172:9-14

## Minor points

### Abstract

#### • Line 5 : The abbreviations ENMO and MAD need definitions

**Answer:** We now explain the abbreviations in the abstract.

**Change in manuscript**

Abstract line 2-5: Movement behavior features such as time use estimates, average accelerations and intensity gradients are crucial in understanding associations with cardiometabolic health. The aim of this study was to 1) compare movement behavior features processed by commonly used accelerometer metrics among adults (i.e., Euclidian Norm Minus One (ENMO), Mean Amplitude Deviation (MAD) and counts per minute (CPM)),

#### • Line 9: in what country?

**Answer:** We added the country in the abstract by referring to the Belgian nationality of the participants

**Change in manuscript**

Abstract line 16-18: Movement behavior data from 213 Belgian adults (mean age 45.8±10.8 years, 68.5% female) differed according to the metric used, with ENMO representing the most sedentary movement behavior profile and CPM vector magnitude representing the most active profile.

### Background

#### • Lines 35-41: The ‘combined’ guidelines mentioned here are new and I’m not sure if they extend beyond Canada - most countries and the WHO only have guidelines for physical activity. I think it’s worth being clear that these are not internationally-agreed guidelines, and in fact the WHO 2020 guidance explicitly says that ‘evidence was insufficient to quantify a sedentary behaviour threshold’.

**Answer**: In the background section, we highlight both WHO guidelines and the Guidelines from the Canadian Society for Exercise Physiology (CSEP). For PA, the guidelines are the same. However, it is correct that the CSEP guidelines have specific recommendations on SB duration which is not mentioned by the WHO guidelines due to insufficient evidence. The SB guideline of the CSEP is mainly based on cross-sectional data which hampers causality. As we want to evaluate the impact of metric application on compliance with the 24-hour movement behavior guidelines, we decided to use the CSEP guidelines for classification of SB compliance. We now mention this limitation in our discussion section. Moreover, we removed the reference of Bull and colleagues (2020) as we only refer to the guidelines of the CSEP.

**Change in manuscript**

Discussion line 482-485: Next, adults were categorized as noncompliant with SB guidelines if they exceeded the threshold of 8 hours per day, as per the the Canadian Society of Exercise Physiology guidelines. However, the use of this specific threshold has been criticised as it is largely underpinned by cross-sectional evidence [2]. Therefore, guideline compliance in this paper should be interpreted with caution.

#### • Lines 61-62: I’m not sure what point is being made here. Regardless of metrics, if the accelerometer is removed, determining non-wear time will be a problem. If not, then determining sleep time is an issue.

**Answer**: In the process of analyzing data using Actilife, the initial step involves manual screening for non-wear and wear time based on predefined time frames within the software application. However, there are examples where the software does not accurately register this distinction, making it challenging to separate non-wear from wear signals within these specified timeframes. This issue can have an impact on consistency when multiple researchers analyze the same dataset, especially when non-wear time relies on the human assessment of this distinction. In this study, GGIR was used which employs a uniform algorithm for detecting non-wear time, ensuring consistency across data files and avoiding human error. This has already been mentioned in the strengths section of the paper. See discussion line 474-476: ***“Perfect reproducibility across the four metrics was ensured by the use of the GGIR package, which allows for consistent data reduction features, i.e., autocalibration, sleep algorithm, and nonwear detection methods.”***

### Methods

#### • Line 89-90: what does ‘active on the job market’ mean? Is this employed, or unemployed and looking for work? (to me, the ‘job market’ refers to recruitment for new posts)

**Answer**: We decided to change this wording to clarify that we only included adults that were employed for at least 50% per week.

**Changes in manuscript**

Methods line 102-103: This cross-sectional study used a Belgian sample of adults aged 25 to 64 years who were employed for at least 50% per week.

#### • Line 97: more details required. How many days? Including weekends? Was there a minimum wear time to be included (either per day, or number of days?)

**Answer:** This part is mentioned in movement behavior feature analysis where we explain how to process the data. See methods line 152-154: ***“Accelerometer data were considered valid if the device provided data for at least four days (including a minimum of three weekdays and one weekend day) with a minimum of 16 valid wear-time hours a day [17].”***

#### • Line 124-5: can you give more detail of the educational system for the international reader eg ages, qualifications. I don’t understand the distinction between ‘higher education degree’ and ‘university degree’.

**Answer**: The distinction between ‘higher educational degree’ and ‘university’ is referring to college degrees and university degrees. We changed ‘higher education degree’ into ‘college degree’.

**Changes in manuscript**

Methods line 137-138: Educational level was classified as low (primary or secondary school degree), middle (college degree) or high (university degree).

#### • Line 136-8: add reference or justification

**Answer**: We added the reference of Migueles and colleagues 2019.

References: 9. Migueles JH, Rowlands AV, Huber F, Sabia S, van Hees VT. GGIR: A Research Community–Driven Open Source R Package for Generating Physical Activity and Sleep Outcomes From Multi-Day Raw Accelerometer Data. Journal for the Measurement of Physical Behaviour. 2019;2(3):188-96.

#### • Lines 142+ this information on metrics belongs in the background section

**Answer:** Based on the suggestion of the first major comment, we added additional information in the background section on the data processing metrics and updated table S1 with additional information on these metrics. However, as there are more metrics and cut-points than used in our paper, we prefer to keep the metric accompanied cut-points in the method section.

#### • Line 149-155 : mixing of reference styles

**Answer:** We changed the references in the text to last name and colleagues [reference number].

**Changes in manuscript**

Methods line 158-169: The cut-points of Hildebrand and colleagues [18] were used, i.e., light PA (LPA) (47 mg), moderate PA (MPA) (*69 mg*) and vigorous PA (VPA) (260 m*g*). Second, as the ENMO metric often suffers from calibration errors being too sensitive, the MAD metric (which works with average subtractions) seems to better account for offsetting signal noise [13]. MAD describes the distance of data points around the mean [20, 21]. The cut-points of Vaha Ypya and colleagues [19,20] were used, i.e., LPA (22.5 m*g*), MPA (94 m*g)*, and VPA (396 m*g*). Third, the new “activity counts” metric, which replicates the Actilife process based on the recently published paper [14]. This metric works with CPM cut-points for the VA, as in Troiano and colleagues [21] , i.e., LPA (100 CPM), MPA (2020 CPM), and VPA (5999 CPM), as well as for VM, by applying the cut-points of Sasaki and colleagues [22,23], i.e., LPA (200 CPM), MPA (2690 CPM) and VPA (6166 CPM).

#### • Line 178: add reference

**Answer:** We added the book reference of Portney 2009.

References: 25. Portney LG. WM. Foundations of Clinical Research: Applications to Practice. 2nd Ed. ed: Pearson/Prentice Hall; 2009. 696 p.

#### • Line 183-185: It’s not clear, but either these are the same MB measures for different metrics, in which case they are measuring the same thing so no surprise if they’re correlated, or they’re the same metric and different MBs, in which case they must be correlated because they sum to 24h. Either way, please justify why correlations are useful/relevant.

**Answer**: The reason for exploring correlations between average acceleration, intensity gradient and time-use estimates was to show the independency of the intensity gradient compared to the cut-point dependent time use estimates. However, upon reflection on the reviewer’s comment, and the fact that this has already been investigated in other papers, we have now removed this part of the paper and added an additional section on the independency of intensity gradient in the discussion.

**Changes in manuscript**

Methods line 198-199: ~~The intercorrelations between the different movement behavior features were explored using Pearson correlations via the R package~~ *~~Corrplot~~* ~~[27].~~

Results line 303-314: ~~Regarding the intercorrelations of both features, average acceleration was strongly correlated with MVPA (ENMO, r= 0.81; MAD, r=0.86; CPM, VA, r=0.87; CPM, VM, r=0.83), while LPA was poorly to moderately correlated with average acceleration (ENMO, r= 0.55; MAD, r= 0.34; CPM, VA, r=0.35; CPM, VM, r= 0.49). The magnitudes of correlation between the intensity gradient and LPA (ENMO, r=0.30; MAD, r= -0.15; CPM, VA, r=-0.26; and CPM, VM, r= -0.42) and MVPA (MAD, r= 0.73; CPM, VA, r=0.82; CPM, VM, r=0.63) were smaller than those mentioned for average acceleration (except for ENMO MVPA, r=0.82). Finally, the average acceleration and intensity gradient were strongly correlated for ENMO, MAD and CPM VA (r=0.80, r=0.77, and r=0.70, respectively) but only moderately correlated for CPM VM (r=0.47) [see Fig. 2].~~

~~Fig. 2. Intercorrelations between single movement behaviors, average acceleration and intensity gradient according to four metrics (ENMO, MAD, CPM VA, CPM VM)~~

~~[insert Fig. 2]~~

Discussion line 432-444: Despite the absence of reference values for hip-worn accelerometer data, the strength of the correlation between average acceleration and intensity, as well as the duration of LPA or MVPA, provides valuable insights into health associations related to both the quantity and intensity of activity [10, 37]. The independence of the intensity gradient compared to cut-point dependent time use at different intensities has previously been reported, evidenced by a smaller magnitude of correlation between the intensity gradient and LPA and MVPA compared to a higher correlation between intensity gradient and average acceleration [10, 40]. Moreover, both the intensity gradient and average acceleration were more strongly associated with MVPA than with LPA, indicating a better capture of higher intensities [10, 40].

#### • Lines 214-7 for log-transformed outcomes, exp(coef) should be reported as % change – it’s not clear whether this is what is proposed

**Answer:** As indicated in the supplementary table legend, all cardiometabolic outcome variables were log-transformed. Thus, it is true that the regression coefficients would refer to % change in the outcome. However, we do not present the individual regression coefficients. Instead, we use the model to make predictions for the log-transformed outcomes, which are then back transformed to their original raw units. In our time-reallocation analyses we compare predictions expressed in their raw units, rather than % change.

**Changes in manuscript**

Methods line 234-244: For the 24h-MB composition the interpretation of the strength and directions of associations are plotted as time reallocations models. These predictions estimated the average difference in cardiometabolic health outcomes when time (e.g., -20 to +20 minutes) in one behavior was proportionally exchanged with time in the remaining behaviors. To enhance the interpretability of the time reallocation models, the log-transformed data were back-transformed to their raw units prior to computing differences. The outcomes of the time reallocations are presented as the absolute differences between the estimated and the mean cardiometabolic variable, and the standardized effect size (ES), which is the absolute difference divided by the standard deviation of the particular variable.

#### • Lines 218, 222: Please avoid the term ‘statistically significant’ and report all estimates with p-values, regardless of significance level.

**Answer:** In the original manuscript, we already reported all estimates with p-values regardless of significance levels but we neglected to mention it in the statistical description. This has been amended accordingly.

**Changes in manuscript**

Methods line 231-239: The estimates, t-value, p-value and adjusted R² were reported for associations between the average acceleration and intensity gradient on the one hand and cardiovascular variables on the other hand.

For the 24h-MB composition, the interpretation of the strength and directions of associations are plotted as time reallocations models. These predictions estimated the average difference in cardiometabolic health outcomes when time (e.g., -20 to +20 minutes) in one behavior was proportionally exchanged with time in the remaining behaviors.

### Results

#### • Fig1: graphs are different sizes and dimensions

**Answer**: We changed the sizes and dimensions to make it more uniform.

**Changes in manuscript**

See Figure 1.

#### • Fig2: I can’t read some of these numbers- rethink colour scheme?

**Answer**: As we decided to remove the part on correlations between movement behavior features, this figure has been deleted.

**Changes in manuscript**

~~Fig. 2. Intercorrelations between single movement behaviors, average acceleration and intensity gradient according to four metrics (ENMO, MAD, CPM VA, CPM VM)~~

~~[insert Fig. 2]~~

#### • Table S5: this table has very little information of use, and none of the % mentioned in the text are reported here.

**Answer:** Table S5 provides the significance levels resulting from pairwise comparisons of the metrics using MANOVA and this is the necessary output to report when working with compositional data analysis. Given the observed significant differences between all compositions (p-value <0.001) derived from the four distinct metrics, we decided to put this information in the supporting information.

The percentages mentioned in the text can be found in Table 1. We added [see table 1] at the end of the section to guiding the reader to the correct table.

**Changes in manuscript**

Results line 315-323: Considering the codependency of behaviors, the compositional analysis showed different results according to the metrics. Significant differences were found between the four metrics for the mean time spent on 24h MBs (all p<0.001) [Supporting Information S5]. Using ENMO resulted in 24h-MB compositions with the highest proportion of SB (59% for ENMO compared to 42% for MAD, 40% for CPM VA and 35% for CPM VM). Regarding the LPA concentration, the CPM VM had the highest percentage of time spent in the LPA (25% for the CPM VM compared to 2% for the ENMO, 17% for the MAD 22% for CPM VA). Finally, the highest proportion of MVPA in a 24h-MB composition was found for MAD (5%), whereas lower proportions were found for ENMO (3%), CPM VA (2%) and CPM VM (4%) [see Table 1].

#### • Table S6: what does the column ‘no guidance’ show?

**Answer:** This refers to the column ‘No guidelines’ which was hidden as the column width was not wide enough. The column size of the ‘No guidelines’ column was changed to make it more clear.

### Discussion

#### • Lines 356-369:.Can you expand on why they’re different eg what aspects of PA different metrics are capturing, when one might be more or less suitable than another?

**Answer**: We understand the need for more guidance on which metric is better then the other. However, as these data reduction metrics include a specific way in subtracting the acceleration signal from the gravitational signal, it is difficult to say which one is preferred and why. The methods to subtract are different and no gold standard exists up until now.

The same reasoning applies for the cut-points to classify activity intensities. A lot of different cut-points are used to classify activity intensities. However, it is difficult to say which one is the best. Compared to the metric choice, more clarity exists on the best cut-point choice based on the method for criterion-based validation such as comparing against metabolic equivalents (METs) or the sample size.

Therefore, in this paper it is difficult to conclude which metric (+ cut-points) is the most appropriate. However, in the discussion, we addressed the more unrealistic SB and LPA classification by the ENMO metric in combination with Hildebrand cut-points.

**Changes in manuscript**

Discussion line 392-413: Differences between metrics were found in determining movement behavior features, including cut-point-related times spent in single movement behaviors and cut-point-independent features such as average acceleration and intensity gradient. The ENMO metric produced the most sedentary time-use profile and average acceleration and intensity gradient, while the CPM VM metric produced the most active time-use profile. CPM VA and MAD demonstrated moderate to good agreement for all the features (time spent in SB and LPA, average acceleration, intensity gradient), except for time spent in MVPA. When considering the codependency of behaviors, all the 24h-MBs compositions were significantly different depending on the metric used. Thus, decisions on which data reduction metric to process accelerometry data can lead to different results and conclusions. As indicated by previous research in children and adults, differences found in time-use estimates are attributable to intensity-based cut-points accompanied by the metric [14, 15, 33-35]. Our study showed that cut-point independent features (i.e., average acceleration and intensity gradient) can also exhibit poor agreement between most metrics. Despite the main advantage of comparability across cohorts and accelerometers, caution is warranted when comparing them across metrics (ENMO, MAD, CPM VA, CPM VM) [10]. As our study did not attempt to validate one method above another, we are unable to make recommendations about metric selection. Nevertheless, in our study, the ENMO metric (Hildebrand and colleagues cut-point [18]) led to the most disparate, and perhaps unrealistic, estimates of SB and LPA.. Future research is necessary to further explore the most appropriate metric and cut-point for hip-worn data.

#### • Line 375-378: a related point is that much of the evidence on which guidelines are based is still from self-report rather than accelerometer PA, and even in the latter case much of it is on older count-based approaches. Does this suggest we should try to use metrics that replicate this evidence even if they are not accurate? Or are the newer metrics presented here ‘better’ in any way? Can you say anything about when estimates of MBs might be over or underestimated?

**Answer**: Utilizing guidelines comes with limitations. One significant drawback is the rigid thresholds that categorize individuals into either compliant or non-compliant groups, where even a one-minute difference can determine whether an individual is classified as a complier or not. The discussion section addresses this drawback (line 419-422): ***“Movement behavior guidelines are considered comprehensible for the general population, but in research, we must be aware of the rigidity of such guidelines and the potential consequences of categorizing individuals based on minute deviations.”***

Secondly, the SB guidelines are primarily formulated based on insights drawn from cross-sectional data. This aspect is addressed within the limitations section of this paper (line 482-485): ***“Next, adults were categorized as noncompliant with SB guidelines if they exceeded the threshold of 8 hours per day, as per the Canadian Society of Exercise Physiology guidelines. However, the use of the specific threshold has been criticised as it is largely underpinned by cross-sectional evidence [2]. Therefore guideline compliance in this paper should be interpreted with caution***.”

Hence, our focus is not on comparing self-reported and objectively measured PA. The aim of the section on guideline compliance is to emphasize the difference in compliers and non-compliers depending on the metrics used (in combination with the selected cut points) . Time-use epidemiology often references health associations between these two groups (see reference 1 Rollo and colleagues 2022) but our study demonstrated that the selection of the data processing metric accompanied with cut-points results in different conclusions regarding the prevalence of adults complying with guidelines. The novel features, such as average accelerations and intensity gradients, offer insights into the overall activity patterns without relying on specific cut-points. These features serve as supplementary information when describing 24h-MBs attributes, providing additional details beyond the classification into different activity intensities.

In this study, we refrain from determining which metric (+cut-points) overestimates or underestimates, as there is no gold standard for comparison. Future investigations could involve direct observation to identify which metric and corresponding cut-points offer the most accurate estimates of movement behaviors. Subsequent research is shifting towards Machine Learning approaches to analyze activity patterns, as this can handle much more than the average time spent in the different behaviors. This gives the opportunities to handle complex movement behavior patterns by for example combining accelerometer data with heart rate variability which can classify activities accurately. Moreover this can be trained to take into account individual variability. While this topic is beyond the scope of our current paper, we added this as a recommendation for future research.

**Changes in manuscript**

Discussion line 476-485: For each metric a commonly used cut-point was chosen to classify activity intensities. Although commonly used cut-points were selected, other cut-points are available, which are all based on a specific validation protocol in a specific sample. Using other cut-points might provide other results. Future research should focus on newer Machine Learning Techniques to classify movement behavior patterns, which can align accelerometer data with for example heart rate variability to accurately classify activities [47]. Next, adults were categorized as noncompliant with SB guidelines if they exceeded the threshold of 8 hours per day, as per the Canadian Society of Exercise Physiology guidelines. However, the use of the specific threshold has been criticised as it is largely underpinned by cross-sectional evidence [2]. Therefore guideline compliance in this paper should be interpreted with caution.

References: 47. Farrahi V, Rostami M. Machine learning in physical activity, sedentary, and sleep behavior research. Journal of Activity, Sedentary and Sleep Behaviors. 2024;3(1):5.

#### • Line 388-391: what valuable insights?

**Answer:** With valuable insights we mean additional insights. The novel features, such as average accelerations and intensity gradients, offer insights into the overall activity patterns without relying on specific cut-points. These features serve as supplementary information when describing 24-hour movement behaviors attributes, providing additional details beyond the classification into different activity intensities.

**Changes in manuscript**

Discussion line 432-444: Despite the absence of reference values for hip-worn accelerometry data, the strength of the correlation between average acceleration and intensity, as well as the duration of LPA or MVPA, provides additional insights into health associations related to both the quantity and intensity of activity [10, 37]. The independence of the intensity gradient compared to cut-point dependent time use at different intensities has previously been reported, evidenced by a smaller magnitude of correlation between the intensity gradient and LPA and MVPA compared to a higher correlation between intensity gradient and average acceleration [10, 40]. Moreover, both the intensity gradient and average acceleration were more strongly associated with MVPA than with LPA, indicating a better capture of higher intensities [10, 40].

#### • Line 391-397: what are the implications of this? Are you arguing that some metrics are better than others? Suitable for different purposes?

**Answer:** As mentioned previous comments, the is not possible to determine which metric is better which is also not the aim of the study. As these metrics include different ways of separating the acceleration signal from the gravitational signal, it is not straightforward which metric is better. Nevertheless, GGIR uses the ENMO metric as default and as stated in the GGIR vignette this is a pragmatic choice as there is no superiority of any metric. However, it is known that the MAD metric is less sensitive for calibration errors and this is the reason why some researchers prefer this metric. These considerations are already mentioned in the manuscript.

See background line 62-66: ***“Commonly used data reduction metrics for processing raw accelerometer data are the Euclidian Norm Minus One (ENMO) and Mean Amplitude Deviation (MAD) as these analytic techniques are perceived as not too complex for users as well they have the ability of quantifying output in universal units instead of abstract scales [see Supporting Information S1 for more details].”***

See methods line 155-161: “***First, the ENMO metric (default metric in GGIR) was used to analyze the raw accelerometer data. The ENMO is calculated from the resultant vector of the measured orthogonal acceleration (three raw acceleration signals), adjusted for gravity by subtracting one gravitational unit and rounding to zero [8, 12, 18]. The cut-points of Hildebrand and colleagues [18] were used, i.e., light PA (LPA) (47 mg), moderate PA (MPA) (69 mg) and vigorous PA (VPA) (260 mg). Second, as the ENMO metric often suffers from calibration errors being too sensitive, the MAD metric (which works with average subtractions) seems to better account for offsetting signal noise [12].”***

Next, this part of the discussion refers to the correlations between movement behavior features (time use of SB, LPA, MVPA, average acceleration, intensity gradient). Average acceleration and intensity gradient are not better than time use estimates but we can see this as additional features which say something about the quantity and intensity of activity during the day without relying on cut-points. The advantage of the time use estimates is the classification in different activities but have the disadvantage that they rely on cut-points

**Changes in manuscript**

Background line 62-66: “Commonly used data reduction metrics for processing raw accelerometer data are the Euclidian Norm Minus One (ENMO) and Mean Amplitude Deviation (MAD) as these analytic techniques are perceived as not too complex for users as well they have the ability of quantifying output in universal units instead of abstract scales [see Supporting Information S1 for more details] [8, 9, 12].”

Discussion line 392-413: Differences between metrics were found in determining movement behavior features, including cut-point-related times spent in single movement behaviors and cut-point-independent features such as average acceleration and intensity gradient. The ENMO metric produced the most sedentary time-use profile and average acceleration and intensity gradient, while the CPM VM metric produced the most active time-use profile. CPM VA and MAD demonstrated moderate to good agreement for all the features (time spent in SB and LPA, average acceleration, intensity gradient), except for time spent in MVPA. When considering the codependency of behaviors, all the 24h-MBs compositions were significantly different depending on the metric used. Thus, decisions on which data reduction metric to process accelerometry data can lead to different results and conclusions. As indicated by previous research in children and adults, differences found in time-use estimates are attributable to intensity-based cut-points accompanied by the metric [14, 15, 33-35]. Our study showed that cut-point independent features (i.e., average acceleration and intensity gradient) can also exhibit poor agreement between most metrics. Despite the main advantage of comparability across cohorts and accelerometers, caution is warranted when comparing them across metrics (ENMO, MAD, CPM VA, CPM VM) [10]. As our study did not attempt to validate one method above another, we are unable to make recommendations about metric selection. Nevertheless, in our study, the ENMO metric (Hildebrand and colleagues cut-point [18]) led to the most disparate, and perhaps unrealistic, estimates of SB and LPA.. Future research is necessary to further explore the most appropriate metric and cut-point for hip-worn data.

#### • Line 398-418: again, this is just reporting results – can you explain what this means for choosing different metrics?

**Answer:** We tried to shorten this paragraph where we added the implications of choosing for a specific metric. However, as mentioned before it is not possible with these results to point out which metric is the best one to choose.

**Changes in manuscript:**

Discussion line 445-470: While the general trends in associations between cut-point dependent 24h-MB composition and cardiometabolic health were similar across all metrics, the strength of the associations and type of behavior involved in the associations varied depending on the metric used. The ENMO metric showed the most associations with different cardiometabolic health variables (BMI, WC, HbA1c and fat%), whereas the MAD was associated with only WC and fat% and the CPM VM was associated with only fat%. Moreover, MVPA predicted the greatest improvement across metrics. These results are comparable to those of a recent review highlighting the greatest health effects of reallocating time toward MVPA, where evidence for reallocating time into LPA as well as out or into sleep is more inconclusive [44]. In this study, the composition retrieved from the ENMO metric seemed to have unrealistic results regarding LPA and SB which in turn might affect the associations. Furthermore, differences between metrics were found for associations with cut-point independent features (average acceleration and intensity). All the metrics showed significant associations with the intensity gradient and BMI, but again the directions of association were different. As studies reporting the association between these newer cut-point independent movement behavior features and cardiometabolic health are limited [45, 46], this paper emphasizes the influence of the selected metrics when comparing results with other research.

### Conclusion

#### • Line 435-437: can you offer a bit more guidance than just awareness? Surely reporting analyses using multiple metrics will just be confusing and encourage cherry-picking of results – what we need is guidance on which metric to use, and possibly when.

**Aswer**: We understand that more guidance on the preferred metrics is needed but this is difficult as no gold standard exist. A recommendation we can make is highlighting that the metric shows less realistic results compared to the other metrics (very low LPA estimates e.g. 34 min/day). We added this remark in the conclusion.

Next, we agree with the comment on our last sentence where we suggest to use multiple metrics. Therefore we removed this sentence and future research has been commented in the discussion section. See discussion line 479-482. ***“Future research should focus on newer Machine Learning Techniques to classify movement behavior patterns,*** ***which can align accelerometer data with for example heart rate variability to accurately classify activities [47].”***

**Changes in manuscript**

Conclusion line 490-497: Depending on the chosen metric, differences were found for cut-point dependent (time use estimates) and cut-point independent (average acceleration and intensity gradient) movement behavior features. The ENMO metric classified adults with the most sedentary behavior profiles as CPM VM had the highest activity profiles, both with implications for guideline compliance prevalence and associations with cardiometabolic health. However classification of SB and LPA seemed the least realistic when the ENMO metric was used. Researchers should be aware of the implications of metric choice in data processing for data interpretation and comparability across studies.
